# A Proton-Coupled Transport System for β-Hydroxy-β-Methylbutyrate (HMB) in Blood–Brain Barrier Endothelial Cell Line hCMEC/D3

**DOI:** 10.3390/nu13093220

**Published:** 2021-09-16

**Authors:** Kei Higuchi, Sathish Sivaprakasam, Souad R. Sennoune, Jiro Ogura, Yangzom D. Bhutia, Ricardo Rueda, Suzette L. Pereira, Vadivel Ganapathy

**Affiliations:** 1 Department of Cell Biology and Biochemistry, Texas Tech University Health Sciences Center, 3601 4th Street, Lubbock, TX 79430, USA; higuchi@toyaku.ac.jp (K.H.); sathish.sivaprakasam@ttuhsc.edu (S.S.); souad.Sennoune@ttuhsc.edu (S.R.S.); jiro.ogura@med.id.yamagata-u.ac.jp (J.O.); yangzom.D.Bhutia@ttuhsc.edu (Y.D.B.); 2 Abbott Nutrition, Research & Development, 18004 Granada, Spain; ricardo.rueda@abbott.com; 3 Abbott Nutrition, Research & Development, Columbus, OH 43219, USA; suzette.pereira@abbott.com

**Keywords:** β-hydroxy-β-methylbutyrate, MCT1 (SLC16A1), MCT4 (SLC16A3), LAT1 (SLC7A5), mTOR, blood–brain barrier

## Abstract

β-Hydroxy-β-methylbutyrate (HMB), a leucine metabolite, is used as a nutritional ingredient to improve skeletal muscle health. Preclinical studies indicate that this supplement also elicits significant benefits in the brain; it promotes neurite outgrowth and prevents age-related reductions in neuronal dendrites and cognitive performance. As orally administered HMB elicits these effects in the brain, we infer that HMB crosses the blood–brain barrier (BBB). However, there have been no reports detailing the transport mechanism for HMB in BBB. Here we show that HMB is taken up in the human BBB endothelial cell line hCMEC/D3 via H^+^-coupled monocarboxylate transporters that also transport lactate and β-hydroxybutyrate. MCT1 (monocarboxylate transporter 1) and MCT4 (monocarboxylate transporter 4) belonging to the solute carrier gene family SLC16 (solute carrier, gene family 16) are involved, but additional transporters also contribute to the process. HMB uptake in BBB endothelial cells results in intracellular acidification, demonstrating cotransport with H^+^. Since HMB is known to activate mTOR with potential to elicit transcriptomic changes, we examined the influence of HMB on the expression of selective transporters. We found no change in MCT1 and MCT4 expression. Interestingly, the expression of LAT1 (system L amino acid transporter 1), a high-affinity transporter for branched-chain amino acids relevant to neurological disorders such as autism, is induced. This effect is dependent on mTOR (mechanistic target of rapamycine) activation by HMB with no involvement of histone deacetylases. These studies show that HMB in systemic circulation can cross the BBB via carrier-mediated processes, and that it also has a positive influence on the expression of LAT1, an important amino acid transporter in the BBB.

## 1. Introduction

β-Hydroxy-β-methylbutyrate (HMB) is an endogenous minor metabolite of the essential amino acid leucine, a branched-chain amino acid like valine and isoleucine. The metabolism of all three branched-chain amino acids occurs robustly not only in the liver, but also in the skeletal muscle, brain, and retina [1,2,3]. There are several pathways for the metabolism of leucine, and only <10% of leucine goes through the pathway that produces HMB. As such, the circulating levels or tissue levels of HMB are in the low micromolar range under normal physiological conditions. Interestingly, this minor metabolite has been found to have a significant impact on the cellular signaling pathways associated with anabolic metabolism, but only at supra-physiological concentrations [4,5,6]. The most notable of these pharmacological effects of HMB is the stimulation of protein synthesis in skeletal muscle via the activation of signaling pathways involving phosphoinositide-3-kinase (PI3K), mitogen-activated protein kinases (MAPK), extracellular signal-regulated kinases (ERK1), and mTOR (mechanistic target of rapamycin). Therefore, HMB is used at supra-physiological (or therapeutic) doses as a nutritional ingredient to improve muscle health, particularly for conditions such as sarcopenia and muscle wasting. It is also used by athletes to enhance muscle mass and strength. A recent report has shown that HMB also protects against diet-induced obesity; interestingly, the mechanism responsible for this phenomenon is HMB-induced alterations in colonic bacteria, consequently increasing the luminal concentrations of the short-chain fatty acid propionate [7]. Propionate is a known inhibitor of histone deacetylases [8,9] and also an agonist for the G-protein-coupled receptor GPR43 [10,11]. The anti-obesity effect of orally administered HMB might arise from the influence of bacteria-generated propionate on organs such as the intestinal tract, liver and adipocytes.

The biological effects of HMB are not restricted to the peripheral tissues. Orally administered HMB has been shown to have a broad spectrum of beneficial effects in the brain, as evident from in vitro and in vivo studies [12,13,14]. In vitro, HMB promotes differentiation and neurite outgrowth in the mouse neuroblastoma cell line Neuro2a in culture [12]. In vivo, HMB prevents the age-related loss of dendrites in pyramidal neurons in rats [13], and also ameliorates age-related decline in cognitive performance in rats [14]. As in vivo effects are seen with HMB administered to the animals orally, it is inferred that HMB is able to cross the blood–brain barrier (BBB), but there have been no published reports in the literature describing the transport of HMB in BBB endothelial cells. Recently, we reported that HMB is transported into skeletal muscle cells via the H^+^-coupled monocarboxylate transporters MCT1 (monocarboxylate transporter 1) and MCT4 (monocarboxylate transporter 4) [15]. BBB endothelial cells express H^+^-coupled monocarboxylate transporters [16,17,18] and play a key role in the transport of physiologically important monocarboxylates such as lactate and β-hydroxybutyrate (a ketone body), which are transferred from the systemic circulation into the brain across the BBB and function as alternative energy substrates to neurons when glucose supply is limited [19,20]. Therefore, we hypothesized that the same transporters in BBB endothelial cells are also involved in the cellular uptake of HMB, a monocarboxylate structurally similar to lactate and β-hydroxybutyrate. We tested this hypothesis in vitro using the human brain microvascular endothelial cell line hCMEC/D3.

HMB is also known for its intracellular actions as a signaling molecule. Its ability to promote anabolism is related to mTOR signaling [4,5,6]. This suggests that HMB might potentially elicit changes in the transcriptomic profile in target cells. As such, we examined the impact of HMB on the expression of selective transporters in hCMEC/D3 cells.

## 2. Materials and Methods

### 2.1. Materials

The human cerebral microvascular endothelial cell line hCMEC/D3 was purchased from EMD Millipore-Sigma (Burlington, MA, USA). The human breast cancer cell line MCF7 and mouse skeletal muscle cell line C2C12 were obtained from the American Type Culture Collection (ATCC, Manassas, VA, USA). Cell culture media, except for the one used for culturing hCMEC/D3 cells, were obtained from Corning Cellgro (Manassas, VA, USA) and the fetal bovine serum (FBS) was from Atlanta Biologicals (Norcross, GA, USA). L-[^14^C(U)]-Lactate (specific radioactivity, 150 mCi/mmol) was purchased from Perkin Elmer (Waltham, MA, USA). [1-^14^C]-β-Hydroxy-β-methylbutyrate was custom-synthesized by the American Radiolabeled Company (St. Louis, MO, USA). The MCT1-specific inhibitor SR13800 (2,6-dihydro-7-[(3-hydroxypropyl)thio]-2-methyl-4-(2-methylpropyl)-6-(1-naphthalenylmethyl)-1H-pyrrolo[3,4-d]pyridazin-1-one) and the MCT4-specific inhibitor Bindarit were obtained from EMD Millipore-Sigma and Advanced ChemBlocks Inc (Burlingame, CA, USA), respectively. All other reagents were from Thermo Fisher Scientific (Waltham, MA, USA), Sigma Aldrich (St. Louis, MO, USA), or Tokyo Chemical Industry (Tokyo, Japan).

### 2.2. Cell Lines and Culture Conditions

The hCMEC/D3 cells were maintained in EndoGRO medium supplemented with 5% FBS, 0.2% EndoGRO-LS supplement, 5 ng/mL rhEGF, 10 mM L-glutamine, 1 μg/mL hydrocortisone hemisuccinate, 0.75 U/mL heparin sulfate, and 50 μg/mL ascorbic acid. MCF7 and C2C12 cells were cultured in DMEM medium supplemented with 10% FBS, 100 mU/mL penicillin and 100 μg/mL streptomycin.

### 2.3. RT-PCR

Total RNA was extracted from cells using TRIzol Reagent (Thermo Fisher Scientific), and the RNA was reverse transcribed using a high-capacity cDNA reverse transcription kit (Thermo Fisher Scientific) according to the manufacturer’s protocol. PCR and quantitative PCR were performed with Takara Taq Hot Start Version (TaKaRa Biotechnology, Shiga, Japan) or Power SYBR Green PCR master mix (Thermo Fisher Scientific) as described previously [15]. Primer sequences are shown in Table 1. The relative mRNA expression was determined by the 2^−ΔΔCt^ (2^−delta delta Ct^) method. HPRT was used as a housekeeping gene for normalization.

### 2.4. siRNA Transfection

hCMEC/D3 cells were seeded in 24-well culture plates at a density of 2 × 10^5^ cells/well with culture medium. siRNAs for SLC16A1 (solute carrier gene family 16, member A1)/MCT1 (monocarboxylate transporter 1) (Silencer select siRNA, s579), SLC16A3/MCT4 (Silencer select siRNA, s17417), SLC16A4/MCT5 (Silencer select siRNA, s17413 and s17414), SLC16A6/MCT7 (Silencer select siRNA, s17407 and s17408), SLC16A13/MCT13 (Silencer select siRNA, s47339 and s47341) and negative control (Silencer select negative control siRNA) were obtained from Thermo Fisher Scientific. siRNAs were transfected using RNAiMax (Thermo Fisher Scientific) 24 h and 72 h after seeding. Each siRNA, diluted in Opti-MEM I Reduced Serum medium, and RNAiMax were mixed gently and incubated at room temperature for 20 min. These mixtures were added to each well. The cells were incubated for a total of 4 days at 37 °C under 5% CO_2_ and then used for experiments.

### 2.5. Uptake Measurements

hCMEC/D3 cells were seeded in 24-well cell culture plates coated with Collagen type I at a density of 2.0 × 10^5^ cells/well with culture medium. On the day of uptake measurement, the culture plates were kept in a water bath at 37 °C. The medium was aspirated and washed with uptake buffers (for composition, see below). The uptake medium (250 μL) containing radiolabeled L-lactate or radiolabeled HMB in indicated uptake buffers was added to the cells. Following incubation for indicated time periods, the medium was removed by aspiration and the cells were washed three times with ice-cold uptake buffer. The cells were then lysed in 1% sodium dodecyl sulfate/0.2 N NaOH and used for measurement of radioactivity. The Na^+^-containing buffer (140 mM NaCl, 5.4 mM KCl, 1.8 mM CaCl_2_, 0.8 mM MgSO_4_, and 5 mM D-glucose, 25 mM Hepes/Tris (pH 7.5)) was used to evaluate the Na^+^-dependent uptake. The Na^+^-free buffer (the same components except for the equimolar replacement of NaCl with *N*-methyl-d-glucamine chloride (pH 6)) was used to evaluate the H^+^-dependent uptake. The kinetic parameters of the uptake process were determined using the substrate concentration versus the uptake rate relationship according to the Michaelis–Menten equation describing a single saturable transport system. An Eadie–Hofstee plot was used as the linear transformation of the Michaelis–Menten equation to calculate the kinetic parameters.

In experiments related to inhibition of HMB uptake, the substrates of MCTs (lactate, pyruvate, acetate, propionate, butyrate, β-hydroxybutyrate, nicotinate and probenecid), organic anion transporters (*p*-aminohippurate and probenecid), and amino acid transporters (glycine, proline, and leucine) were used. NaCl was added to the uptake buffer as an osmotic control while using some of these inhibitors at 20 mM.

### 2.6. Intracellular pH (pH_in_) Measurements

Cells were grown onto rectangular coverslips (9 × 22 mm) until they reached confluency. Then, the cells were incubated with 7.5 μM SNARF-1-AM in NaCl Buffer, pH 7.4 for 30 min at 37 °C, followed by 30 min incubation in dye-free buffer to ensure the complete hydrolysis of the ester and the leakage of uncleaved dye. Two coverslips were placed back to back in a holder perfusion device and perfused at a rate of 3 mL/min, and the fluorescence of SNARF-1 was monitored with a SLM-8100/DMX spectrofluorometer (Spectronics Instruments, Rochester, NY, USA) equipped for sample perfusion at 37 °C. The sample temperature was maintained at 37 °C by keeping both the water jacket and perfusion buffer at 37 °C using an iso-temperature immersion circulator water bath. All measurements were performed using 4 nm bandpass slits and an external rhodamine standard as a reference. Fluorescence was monitored in continuous-acquisition mode using an excitation wavelength of 534 nm and monitoring emissions at 584, 600, and 644 nm. The fluorescence emission at 584 nm decreases, and that at 644 nm increases, respectively, with increasing pH. The ratio of 644 to 584 nm was used to monitor pH changes. The 600 nm wavelength, which is insensitive to pH, was used to evaluate the efficiency of dye loading, quenching, or other artifacts. Fluorescence data were converted to ASCII format for subsequent analyses using SigmaPlot.

For the in situ calibration of intracellular pH, the cells attached to coverslips were perfused with High K^+^ buffer (10 mm NaCl, 146 mm KCl, 10 mm HEPES, 10 mm MES, 10 mm Bicine, 2 μm valinomycin, 6.8 μm nigericin, 5 mm glucose, pH 5.5–8.0, at intervals of 0.2 pH unit). The buffer contained high K^+^ to approximate the intracellular K^+^ concentration. Nigericin is an ionophore that exchanges H^+^ and K^+^ across the membrane, rendering the pH_in_ equal to the extracellular pH (pH_ex_). Valinomycin is an ionophore that moves K^+^ across the plasma membrane and, together with nigericin, helps to equilibrate pH_in_ and pH_ex_. The ratios (*r* = 644/584) of SNARF-1 were converted to pH using a modified Henderson–Hasselbalch equation [21]. The equation was solved using nonlinear least squares analysis with SigmaPlot to obtain the values of p*K_a_*, *R*_min_, and *R*_max_ for SNARF-1 in these cells. These parameters were used to convert ratio values to pH_in_.

### 2.7. Western Blot

Whole-cell protein lysates were prepared from cells grown at 80–90% confluency using RIPA lysis buffer with a protease–phosphatase inhibitor cocktail. The total protein contents of the samples were quantified using a BCA Protein Assay kit. Lysates were prepared at a final protein concentration of 1 mg/mL, which also contained β-mercaptoethanol. Lysates were heated at 95 °C for 3 min, and then separated by electrophoresis on SDS–PAGE and transferred to a Polyvinylidene difluoride membrane at 70 V for 2 h at 4 °C. After transferal, the membranes were blocked with 5% milk or 5% bovine serum albumin (for phospho-protein) for 1 h and incubated with specific primary antibodies (Cell Signaling; p-mTOR; cat. no. 5536, mTOR; cat. no. 2983, p-p70S6K; cat. no. 9204, p70S6K; cat. no. 9202, Acetyl-Histone H3; cat. no. 9649, Histone H3; cat. no. 4499) overnight at 4 °C with gentle shaking. After incubation, the membranes were washed three times with TBST, 10 min each time, then incubated with HPR-conjugated secondary antibodies (cat. no. 1706515, Bio-Rad Laboratories, U.S.A.) for another hour, washed three times with TBST as before, and bands were visualized using Pierce ECL Western blotting substrate (Thermo Scientific; cat. no. 32106). The experiments were repeated thrice with three separate cell cultures and treatments, and densitometric scanning was used to quantify the signals.

### 2.8. Statistics

Uptake measurements were made in triplicate and the experiments were repeated twice with separate cultures. In all cases, data are expressed as means ± S. D. Statistical differences between control groups and experimental groups were analyzed by paired Student’s t test or by one-way analysis of variance (ANOVA) followed by Dunnett’s test for single and multiple comparisons; a *p* < 0.05 was considered significant.

## 3. Results

### 3.1. H^+^-Coupled HMB Transport in hCMEC/D3 Cells

**Figure 1:**
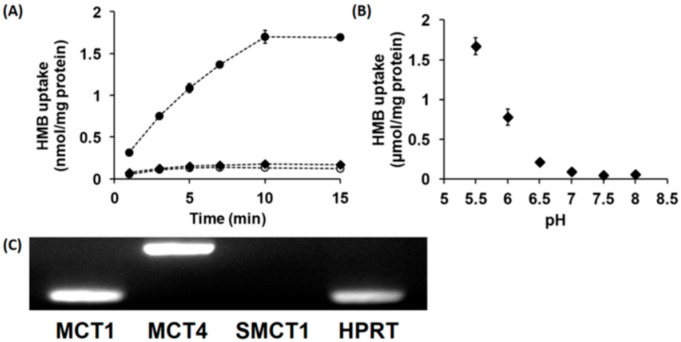
Time- and pH-dependent uptake of HMB and mRNA expression of monocarboxylate transporters in hCMEC/D3 cells. (**A**) Uptake of [^14^C]-HMB (15 μM) was measured for varying time periods in a Na^+^-free (NMDG chloride) buffer at pH 6.0 (●) or 7.5 (○) or NaCl buffer at pH 7.5 (♦). (**B**) The uptake of [^14^C]-HMB (30 μM) was measured for 3 min in a Na^+^-free buffer at pH 5.5–8.0. (**C**) RT-PCR analysis of mRNAs for various monocarboxylate transporters in hCMEC/D3 cells using human-specific primers. HMB, β-hydroxy-β-methylbutyrate; NMDG, *N*-methyl-d-glucamine.

In vitro studies with cloned transporters expressed heterologously in *Xenopus laevis* oocytes or mouse muscle cell line C2C12 in culture have shown that HMB is a transportable substrate for the Na^+^-coupled monocarboxylate transporter SMCT1 (SLC5A8) and the H^+^-coupled monocarboxylate transporters MCT1 (SLC16A1) and MCT4 (SLC16A3) [15]. Therefore, we examined whether HMB is taken up into hCMEC/D3 cells by SMCT1 and/or MCTs. The uptake of HMB in these cells was found to be completely Na^+^-independent with no change in uptake when NaCl in the uptake buffer was replaced completely with *N*-methyl-d-glucamine chloride (Figure 1A). However, in Na^+^-free buffer, reducing the pH from 7.5 to 6.0 markedly stimulated HMB uptake (Figure 1B), indicating the presence of a H^+^-coupled uptake mechanism. We then examined HMB uptake at different pH levels in a Na^+^-free buffer. The uptake showed robust dependence on the concentration of H^+^ in the uptake buffer; the uptake increased more than 10-fold when the pH was changed from 7 to 6. Since a previously published report implicated MCT1, MCT4, and SMCT1 in HMB uptake in muscle cells [15], we examined the expressions of these three transporters in hCMEC/D3 cells (Figure 1C). These studies provided evidence for the expression of MCT1 and MCT4, but not SMCT1, in these cells, thus corroborating the absence of a Na^+^-dependent uptake mechanism but the presence of H^+^-coupled uptake in these cells.

### 3.2. Kinetics of HMB Uptake in hCMEC/D3 Cells

**Figure 2:**
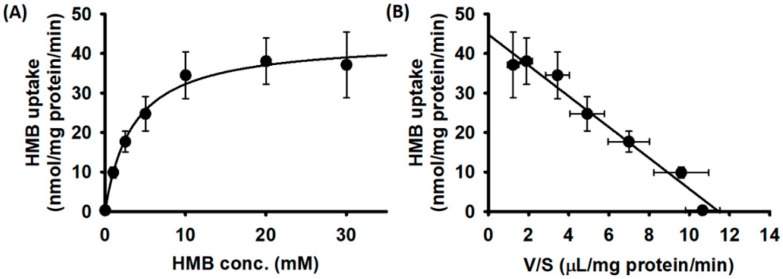
Saturation kinetics of HMB uptake in hCMEC/D3 cells. (**A**) [^14^C]-HMB (30 μM) was used as a tracer to monitor HMB uptake and unlabeled HMB was used to alter HMB concentrations. Uptake measurements were made in a Na^+^-free buffer, pH 6. Data are presented as the HMB concentration (S) versus HMB uptake (V). (**B**) Eadie–Hofstee transformation of the same data (V versus V/S).

We then examined the substrate saturation kinetics for HMB uptake in hCMEC/D3 cells. The uptake data fit best for a transport model involving a single carrier-mediated process and a non-saturable diffusion component. The theoretically calculated diffusional portion was subtracted from the total uptake to determine the portion of uptake that occurred solely via a carrier-mediated transport system. The data given in Figure 2A show that HMB uptake in hCMEC/D3 cells was carrier-mediated and saturable. The linear transformation of the carrier-mediated component of HMB uptake (Eadie–Hofstee plot) was used to determine the values for the kinetic parameters (Figure 2B). The Michaelis constant (Kt) for the uptake process was 3.8 ± 0.7 mM and the maximal velocity (Vmax) was 44.8 ± 2.0 nmol/mg protein/min.

### 3.3. Non-Involvement of OATs and PAT1 (SLC36A1) in HMB Uptake in hCMEC/D3 Cells

HMB is a monocarboxylate and thus an anion; as such, it can potentially function as a substrate for the organic anion transporters (OATs), which transport anionic substrates without coupling to H^+^ [22]. Therefore, we tested the effect of *p*-aminohippurate (PAH), the prototypical substrate for OATs, on the uptake of HMB in hCMEC/D3 cells. We found no effect (Table 2), indicating that HMB uptake in these cells does not involve OATs. In contrast to PAH, the anion probenecid interacts with OATs as well as H^+^-coupled anion transporters, such as the members of the MCT/SLC16 family [23]. Based on this, we examined the effect of probenecid on HMB uptake. We found this organic anion to be an effective inhibitor of HMB uptake in hCMEC/D3 cells. PAT1 (proton-coupled amino acid transporter 1, SLC36A1) is another transporter, widely expressed in normal tissues, whose function is coupled to the transmembrane H^+^ gradient [24,25]. Even though zwitterionic amino acids such as glycine and proline are the prototypical substrates for PAT1, short-chain fatty acids, such as acetate, propionate and butyrate, are recognized as substrates by this transporter; the transport mechanism is an electroneutral H^+^/anion cotransport [26]. This prompted us to examine the possible participation of PAT1 in HMB uptake in hCMEC/D3 cells by monitoring the effect of amino acid substrates of PAT1 on HMB uptake. We found little or no inhibition (Table 2), indicating the minimal involvement, if any, of PAT1 in HMB uptake in these cells.

### 3.4. Substrate Selectivity of the Transport System Responsible for HMB Uptake in hCMEC/D3 Cells

We monitored the substrate selectivity of the transport system that is responsible for HMB uptake in hCMEC/D3 cells by assessing the ability of other monocarboxylates to compete with HMB and inhibit its uptake. We found that all seven monocarboxylates tested in the present study caused marked inhibition of HMB uptake (Table 2). We already know that the transport system interacts with its monocarboxylate substrate HMB with low affinity, based on the Kt value in the low millimolar range. Accordingly, the other monocarboxylates examined in the study also interacted with the transport system with low affinity, as evident from the need for 20 mM of these substrates to cause a ~75% inhibition of HMB uptake.

### 3.5. Relative Contribution of MCT1 and MCT4 to Total HMB Uptake in hCMEC/D3 Cells

**Figure 3:**
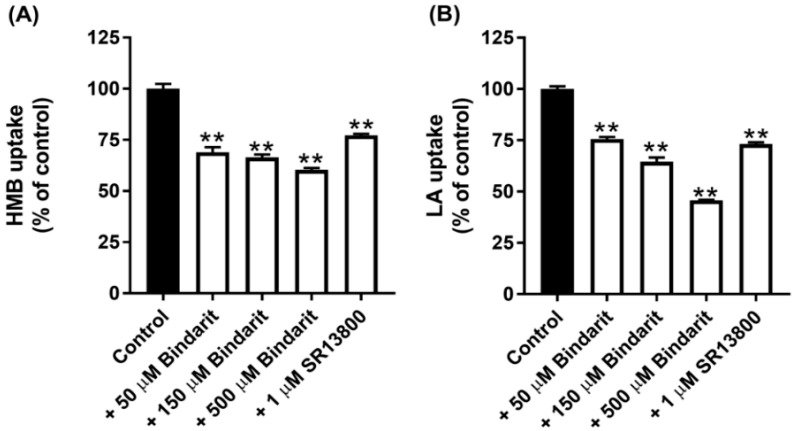
Effect of the MCT1-selective inhibitor SR13800 and MCT4-selective inhibitor Bindarit on the uptake of HMB and lactate in hCMEC/D3 cells. Uptake of HMB (30 μM) (**A**) and lactate (10 μM) (**B**) was measured in a Na^+^-free buffer, pH 6.0, using the respective radiolabeled compounds in the presence of SR13800 (1 μM) or Bindarit (50, 150, and 500 μM). ** *p* < 0.01 compared to the corresponding control uptake measured in the absence of the inhibitor. HMB, β-hydroxy-β-methylbutyrate; LA, lactic acid.

All of the known H^+^-coupled monocarboxylate transporters belong to the solute carrier gene family SLC16. This family has 14 members, but only 5 of them, namely, MCT1-4 and MCT7, have been shown to transport lactate and/or β-Hydroxybutyrate [16]. There are selective inhibitors for two of these transporters: SR13800 for MCT1 [27] and Bindarit for MCT4 [28]. With these inhibitors at concentrations that are expected to block the transport function of the respective MCTs completely, we assessed the contribution of MCT1 and MCT4 to the total uptake of HMB. The data from these experiments (Figure 3A) led to the conclusion that MCT1 contributes ~25% and MCT4 contributes ~40% to HMB uptake in hCMEC/D3 cells. Comparable results were obtained for lactate uptake, except that the contribution by MCT4 was a little bit higher (Figure 3B). The conclusion from these studies is that, in addition to MCT1 and MCT4, there are other transporters that contribute to HMB uptake in BBB endothelial cells.

### 3.6. Expression Profile of Lactate Transporters and Orphan Transporters in SLC16 Family in hCMEC/D3 Cells

**Figure 4:**
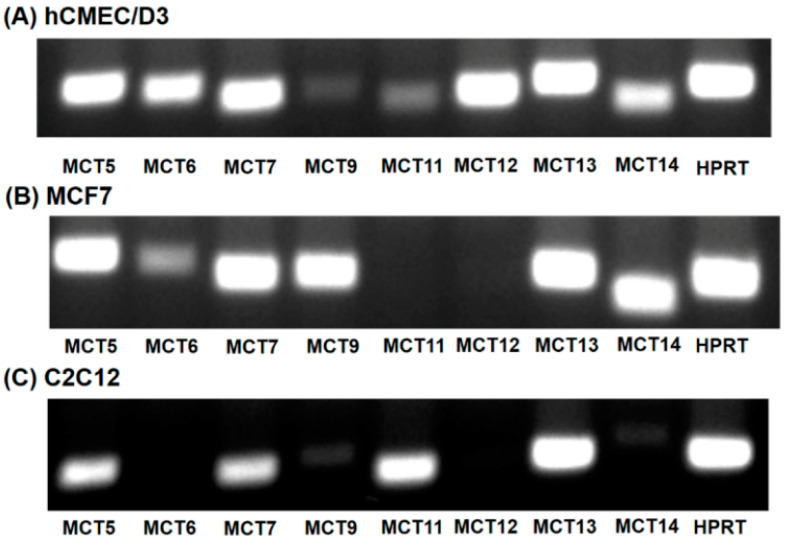
Expression of monocarboxylate transporters in hCMEC/D3 cells (**A**), MCF7 cells (**B**) and C2C12 cells (**C**). RT-PCR analysis of mRNAs for various monocarboxylate transporters was performed using human-specific primers (hCMEC/D3 and MCF7) or mouse-specific primers (C2C12).

Of the 14 members of the SLC16 family, MCT6, MCT8, MCT9 and MCT10 transport substrates structurally unrelated to the small monocarboxylates such as lactate and β-hydroxybutyrate [16]. As the substrate specificity of these four SLC16 family members is known, it is unlikely that any of these transporters mediate HMB uptake. Human MCT2 transports pyruvate but not lactate [16]. MCT3 shows a restricted expression pattern, primarily found in retinal pigment epithelium [29]. To predict which of the members of the SLC16 family, other than MCT1 and MCT4, could contribute to HMB uptake in hCMEC/D3 cells, we performed RT-PCR to determine the expression profile of potential candidates for HMB uptake. For comparison, we also examined the expression profiles in the human breast cancer cell line MCF7 and the mouse skeletal muscle cell line C2C12, both of which exhibit robust H^+^-coupled HMB uptake [15]. These studies showed high expressions of MCT5, MCT6, MCT7, MCT12, MCT13, and MCT14 in hCMEC/D3 cells (Figure 4A). The transporters whose expressions were common among the three cell lines that are positive for HMB transport were MCT5, MCT7, and MCT13 (Figure 4A–C).

**Figure 5:**
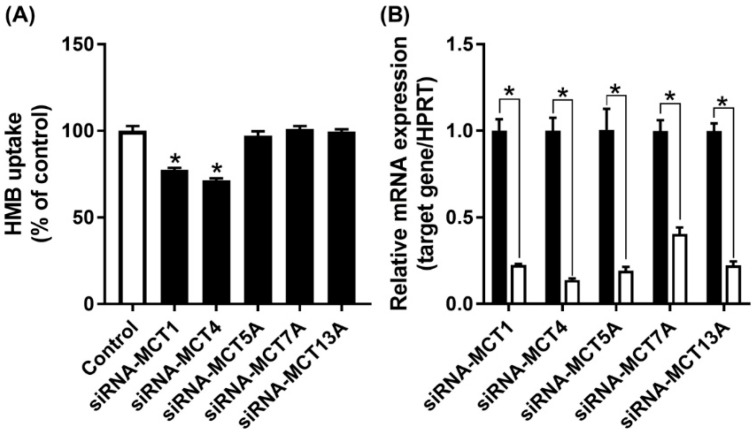
Effect of siRNA-mediated knockdown of MCT transporters on HMB uptake in hCMEC/D3 cells. (**A**) Uptake of HMB (30 μM) in hCMEC/D3 cells treated with MCT transporter-siRNAs was measured in the absence of Na^+^ (NMDG chloride buffer) at pH 6.0. (**B**) mRNA expression of MCT transporters in hCMEC/D3 cells treated with MCT transporter-siRNAs was evaluated by quantitative-RT-PCR. * p < 0.05 compared to corresponding control cells treated with an empty vector in a similar manner (black bars).

MCT7 has been shown to be a transporter specifically for β-hydroxybutyrate [30], while the other two are orphan transporters with no established transport function [16]. As there are no specific inhibitors available for these three transporters, we decided to suppress their expression by siRNA, and then examine HMB uptake to determine if any of these three transporters contributes to HMB uptake in hCMEC/D3 cells. The siRNAs examined in the study did lead to marked reductions in the mRNA levels of the corresponding transporter, but HMB uptake remained unaffected (Figure 5), leading to the conclusion that none of these three transporters make any significant contribution to HMB uptake in hCMEC/D3 cells. In contrast, the siRNA-mediated down-regulation of MCT1 and MCT4 did reduce HMB uptake, corroborating the data from the previous experiments that implicated these two transporters in HMB uptake.

### 3.7. Evidence for Coupled Transport of HMB and H^+^ in hCMEC/D3 Cells

**Figure 6:**
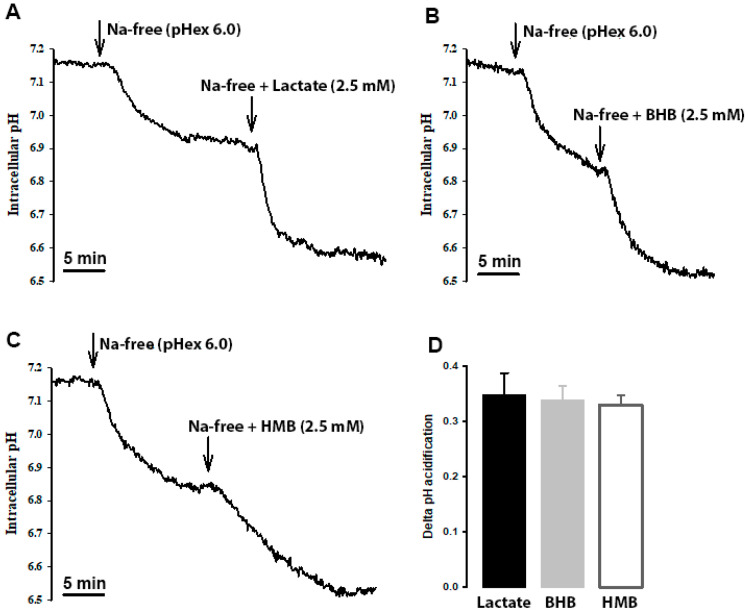
Cells were perfused with Na^+^-containing buffer (pH_ex_ 7.4) until a steady-state pH_in_ was reached. Then, the perfusate was switched to Na^+^-free (pH_ex_ 6.0; arrow), which resulted in a slow acidification of the cell. When the intracellular pH reached a steady state, the perfusate was switched to either (**A**) lactate, (**B**) β-hydroxybutyrate (OH-butyrate) or (**C**) β-hydroxy-β-methylbutyrate (HMB) in Na^+^ free buffer (pH_ex_ 6.0; arrow). In all three cases, a further acidification was observed. The scale bar, in each graph, represents 5 min. The graph bar (**D**) represents the ΔpH_in_ acidification from the Na^+^-free buffer (pH_ex_ 6.0) to either lactate, OH-butyrate or HMB in Na^+^ free buffer (pH_ex_ 6.0). Each treatment was repeated three times. The graph represents the mean ± SEM.

HMB transport in hCMEC/D3 cells is stimulated by extracellular acidic pH, indicating the potential coupled transport of HMB with H^+^. To confirm this mode of HMB transport, we monitored intracellular pH in these cells in the presence of HMB in a Na^+^-free medium, with lactate and β-hydroxybutyrate as positive controls for H^+^-coupled transport. When the cells were switched to Na^+^-free perfusion medium, intracellular pH decreased, indicating cellular acidification (Figure 6). This is because of the transport function of the Na^+^/H^+^ exchanger working in the reverse direction (i.e., Na^+^ efflux coupled to H^+^ influx). When lactate, β-hydroxybutyrate or HMB was added to the Na^+^-free buffer, cellular acidification increased. The magnitude of intracellular acidification was similar for all three monocarboxylates when each was used at 10 mM. These data provide conclusive evidence of the H^+^-coupled transport of HMB in these cells.

### 3.8. Influence of HMB on the Expression of Selective Transporters in hCMEC/D3 Cells and Relevance of mTOR Signaling and HDAC Inhibition to the Process

HMB is a signaling molecule with an ability to activate the mTOR pathway [4,5,6], which controls the expression of the oncogene c-Myc. There is evidence for the induction of the amino acid transporter LAT1 (SLC7A5) by c-Myc [31]. Since LAT1 is the primary amino acid transporter in the blood–brain barrier [32] and its deficiency is associated with decreased levels of specific amino acids (branched-chain amino acids, including leucine) in the brain in the pathogenesis of autism spectrum disorder [33,34], we raised the question as to whether HMB has the ability to enhance the expression of LAT1 in hCMEC/D3 cells.

**Figure 7:**
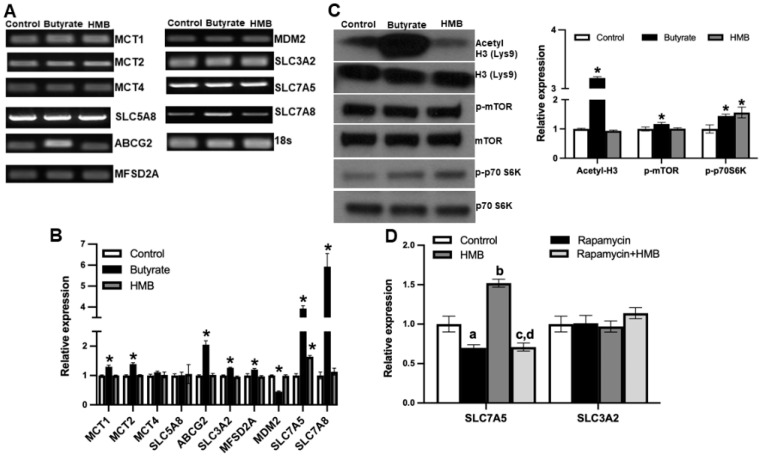
hCMEC/D3 cells were treated with 2 mM of butyrate and 2 mM of HMB for 16 h. (**A**) Gene expression assessed by RT-PCR (**A**), gene expression by quantitative PCR (**B**), and (**C**) Western blot assessment of the total and phospho-form of mTOR and S6 kinase, and also for the total and acetylated form of histone H3 (**C**). hCMEC/D3 cells were treated with rapamycin (10 μM) or HMB (2 mM) alone or in combination for 24 h, and RNA prepared from control and treated cells was used to monitor the expression of SLC7A5 and its subunit SLC3A2 (**D**). Data are represented as mean ± SEM for three independent experiments. * *p* < 0.05. In Figure 7D, a, significantly different from control (*p* < 0.05); b, significantly different from control (*p* < 0.05); c, significantly different from HMB treatment alone (*p* < 0.05); d, not different from rapamycin alone (*p* > 0.05).

To address this question, we treated the cells with 2 mM HMB for 16 h and then prepared RNA and cell lysates. In parallel, we also examined the effects of butyrate (2 mM), a structurally related molecule that is also a signaling molecule but works primarily via the inhibition of class I histone deacetylases, using a similar experimental strategy. Using the RNA from the control and the treated cells, we monitored the expression of mRNAs for nine different transporters by RT-PCR (Figure 7A) and real-time qRT-PCR (Figure 7B). The group of transporters we selected for this study included those involved in the cellular uptake of monocarboxylates, including HMB and butyrate [35] and branched-chain amino acids. Among the nine transporters examined, HMB treatment increased the expression of only SLC7A5. In contrast, butyrate induced the expression of a majority of them. We also monitored the expression of MDM2, an E3 ubiquitin-protein ligase, whose expression runs in parallel with the inhibition of histone deacetylases. MDM2 expression was not altered in HMB-treated cells but was reduced in butyrate-treated cells. This shows that under the conditions and concentrations employed in this experiment, butyrate causes the inhibition of histone deacetylases, but HMB does not. We further probed this differential activation of two different signaling pathways by HMB and butyrate. This was done using the protein lysates prepared from control and treated cells to monitor the acetylation status of histone H3 and the phosphorylation status of mTOR and its downstream target S6 kinase. Butyrate treatment clearly increased the acetylation of histone H3, whereas HMB did not (Figure 7C). In contrast, HMB showed evidence of mTOR activation, as evident from the increased phosphorylation of S6 kinase, even though we could not find any increase in the phosphorylation of mTOR. Butyrate also increased the phosphorylation of mTOR and S6 kinase, but its effect on histone acetylation status was much more robust. These data show that HMB primarily activates the mTOR pathway with little effect on histone deacetylases.

Next, we wanted to determine whether the activation of mTOR was involved in the HMB-mediated induction of the amino acid transporter SLC7A5. Since SLC7A5 heterodimerizes with SLC3A2 to function as an amino acid transporter, we also examined its expression. We used rapamycin, an inhibitor of the mTOR signaling pathway, to establish the involvement of mTOR activation in the HMB-dependent induction of SLC7A5. Treatment with rapamycin slightly decreased the expression of SLC7A5, but more importantly, the HMB-mediated induction of SLC7A5 was blocked completely by the presence of rapamycin (Figure 7D). The effect of HMB was specific to SLC7A5, with no effect on the expression of SLC3A2. These findings lead to the conclusion that the treatment of hCMEC/D3 cells with HMB induces SLC7A5 in association with the activation of mTOR and its downstream targets, but with no involvement of histone deacetylases.

## 4. Discussion

This represents the first study to investigate the uptake of the nutritional ingredient β-hydroxy-β-methylbutyrate (HMB) in blood–brain barrier endothelial cells. The data support the conclusion that the blood–brain barrier has the necessary transport mechanism for the transfer of HMB from systemic circulation into the brain. Previously published studies have shown that orally administered HMB has biological effects on brain function, clearly indicating the accessibility of circulating HMB by the brain [4,5,6,12,13,14]. In the present study, we investigated the transport mechanisms for HMB uptake in blood–brain barrier endothelial cells. These studies have demonstrated that the HMB is taken up by these cells actively, driven by a Na^+^-independent, but H^+^-coupled, transport process. The characteristics of the transport process indicate the predominant contribution of the MCT transporters belonging to the SLC16 family to the uptake process. With specific inhibitors, we were able to demonstrate the involvement of MCT1 and MCT4, but the combined contribution by these two transporters amounts only to ~60% of total uptake. This indicates that there are additional transporters, not yet identified at the molecular level, in the blood–brain barrier that mediate HMB uptake.

The hCMEC/D3 cell line is an established in vitro model for the blood–brain barrier, as this cell line expresses normal endothelial markers CD31, VE cadherin, and von Willebrand factor, forms monolayers in culture, and responds positively to endothelial growth factors [36,37]. Proteomic analyses of this cell line have provided evidence for the expression of MCT1 [38]. The data on MCT4 expression in the blood–brain barrier’s endothelial cells are scarce, and some studies have concluded that MCT4 is not expressed in the blood–brain barrier [39]. In the present study, we found the robust expression of MCT4 in hCMEC/D3 cell line, both at the mRNA level and at the functional level (Bindarit-sensitive uptake). Even though MCT1 and MCT4 are both capable of bidirectional transport, the transport features and substrate affinities have led to the conclusion that MCT1 functions mostly in the influx of its substrates, whereas MCT4 functions in the efflux of its substrates [40,41]. The endothelial cell layer forming the blood–brain barrier is a polarized structure with distinct luminal and abluminal membrane components. The location of MCT1 in the luminal membrane of the blood–brain barrier is known [42], but the molecular identity of the transporter that mediates the efflux of monocarboxylates on the brain side has not been established. MCT4 might fulfill this function in native blood–brain barrier endothelial cells, but this needs to be investigated. It cannot be ruled out, however, that MCT1 functions both in the influx across the luminal membrane and in the efflux across the abluminal membrane of the endothelial cells in this barrier structure. Additional studies are needed to address these issues.

Skeletal muscle is the most predominant tissue target for HMB as a nutritional ingredient. HMB elicits robust anabolic activity in this tissue by promoting protein synthesis mediated by the activation of the mTOR signaling pathway. Since the mTOR pathway is ubiquitous in mammalian tissues, the HMB-mediated activation of this signaling pathway might have biological relevance in blood–brain barrier endothelial cells. With this rationale, we examined the effect of HMB on the phosphorylation status of mTOR and its downstream target S6 kinase in hCMEC/D3 cells. We found evidence of mTOR activation. Since butyrate and β-hydroxybutyrate, two of the endogenous molecules that are structurally similar to HMB, are known inhibitors of histone deacetylases [8,9,43], we investigated the effect of HMB on the acetylation status of histone H3. There was no change in this parameter, suggesting that HMB does not function as an inhibitor of histone deacetylases, at least at the concentration (2 mM) used in the study. Under identical conditions, butyrate showed a robust increase in the acetylation of H3, serving as a positive control for the inhibition of histone deacetylases. We then compared the expressions of selective transporters in control and HMB-treated cells to determine whether the observed mTOR activation has any influence on nutrient transporters, particularly those involved in the transport of monocarboxylates, such as lactate, butyrate, β-hydroxybutyrate, and HMB. There was no effect on the expression of MCTs and SMCT1, nor on the expression of ABCG2, which is known to function as an efflux transporter for monocarboxylates such as butyrate [44]. Then, we examined the expression of SLC7A5 and SLC7A8, two of the amino acid transporters that transport branched-chain amino acids such as leucine. We selected these two transporters solely because leucine is the source of endogenous HMB and SLC7A5 is a known target for mTOR. We found a significant increase in the expression of SLC7A5, but not SLC7A8, in HMB-treated cells. To confirm that the induction of SLC7A5 by HMB involves mTOR signaling, we evaluated the effect of rapamycin, a potent inhibitor of the mTOR pathway. In the presence of this inhibitor, the HMB-mediated increase in SLC7A5 expression was completely blocked. These data show convincingly that the activation of mTOR by HMB is responsible for the observed increase in SLC7A5 expression in these cells. In contrast, butyrate induced the expression of not only SLC7A5, but also SLC7A8, most likely involving the inhibition of histone deacetylases in the process. This finding that HMB induces SLC7A5 expression in the blood–brain barrier’s endothelial cells may have significance because deficiencies in this particular transporter contribute to the pathogenesis of autism spectrum disorder. Mutations in SLC7A5 that lead to the partial loss of transport activity have been found in patients with autism spectrum disorder, associated with a decrease in the brain levels of branched-chain amino acids [33,34]. Since our studies have found that HMB increases the expression of this transporter in the blood–brain barrier, it raises the possibility that HMB as a nutritional ingredient might have therapeutic benefits in patients affected with this disorder.

## Figures and Tables

**Table 1:** Primer sequences used for RT-PCR.

Species	Protein Name	Gene Name	Orientation	Sequence
Human	MCT1	SLC16A1	Forward	GGTGGAGGTCCTATCAGCAG
			Reverse	TCAAGTTGAAGGCAAGCCCA
	MCT4	SLC16A3	Forward	ATCCTGGGCTTCATTGACAT
			Reverse	CTTCAGGAAATGCTCCACCT
	SMCT1	SLC5A8	Forward	CGTCTCTGTGGAACAGTCCT
			Reverse	TAAGACCACCCAGTGTGCAG
	MCT5	SLC16A4	Forward	TCTACCCGGTTTGGGTTCTG
			Reverse	GGGCTCCTGTCCAGTCATAC
	MCT6	SLC16A5	Forward	CCTGAGATCATGTGCCAGTCT
			Reverse	TAGGGCTATTCCAGCCCAGA
	MCT7	SLC16A6	Forward	GGCATCATCTCTGGTCTGGG
			Reverse	TGGTGCGAAAGCAAACACAG
	MCT9	SLC16A9	Forward	CACCCATCGTTGGTTGGTTT
			Reverse	CTTGGGGAGTTGCTTGTTGC
	MCT11	SLC16A11	Forward	GGTTTTCGGTGTACTCCCCG
			Reverse	CTTAGGAAGCCTGACAGGGG
	MCT12	SLC16A12	Forward	ACGGGCAGTCACAAGATGTAT
			Reverse	CAACACTCCCAAGTGGAGCA
	MCT13	SLC16A13	Forward	CAGTTTGGGAGCCCGGTAGG
			Reverse	GAGCCTGACAGCAACCCAAT
	MCT14	SLC16A14	Forward	GATAGTGGGCCCTTTCATCG
			Reverse	CGTTTGCAGCATAGGCACTC
	LAT1	SLC7A5	Forward	CCGTGCCGTCCCTCGTGTTC
			Reverse	GGTTCACCTTGATGGGCCGCT
	LAT2	SLC7A8	Forward	CAGAGTCTGGCCTTCGGCTC
			Reverse	CCAGCCAGAAGTACTCTCCTTTG
	4F2HC	SLC3A2	Forward	CTCGTGGTTCTCCACTCAGG
			Reverse	CCG CAATCAAGAGCCTGTCT
	ABCG2		Forward	TCAGATGGGTTTCCAAGCGT
			Reverse	AACCCCAGCTCTGTTCTGGA
	MFSD2A		Forward	CTCCTGGCCATCATGCTCTC
			Reverse	GGCCACCAAGATGAGAAA
	MDM2		Forward	AGGAGATTTGTTTGGCGTGC
			Reverse	TGAGTCCGATGATTCCTGCTG
	18S		Forward	CCCGTTGAACCCCATTCGT
			Reverse	GCCTCACTAAACCATCCAATCGGTA
Mouse	Mct5	Slc16a4	Forward	ACTCCGTTTCTCTGCAGGTC
			Reverse	AGATCCCAAACCAGGTAGCA
	Mct6	Slc16a5	Forward	TCGTGGTTTCCTTCCATCCTG
			Reverse	CCCAGGCCTGTGATCACTCC
	Mct7	Slc16a6	Forward	GGGGTAATCTCGGGTTTAGGG
			Reverse	TGATAGCTGGTGCGAAAGCA
	Mct9	Slc16a9	Forward	GAGCAGTCTTGCCCCCAATA
			Reverse	ACCCACACTCGAACCTGTTG
	Mct11	Slc16a11	Forward	TATGCCCCACTGGTTTTCGG
			Reverse	TAGGAAGCCTGACAGAGGAGG
	Mct12	Slc16a12	Forward	CGGGCAGTCACCAGATGTA
			Reverse	ACTGACAACACTCCCAAGCG
	Mct13	Slc16a13	Forward	GTTTGGGAGCCCAATAGGCAG
			Reverse	CCTGACAGCAGCCCAATACT
	Mct14	Slc16a14	Forward	TGATCGTGGGACCTTTCATCG
			Reverse	GCAGGTAGGTAAGCCATCCC
Human/Mouse	HPRT		Forward	GCGTCGTGATTAGCGATGATGAAC
			Reverse	CCTCCCATCTCCTTCATGACATCT

**Table 2:** Inhibition of HMB uptake in hCMEC/D3 cells by various anions.

Inhibitor	HMB Uptake (% of Control)
Control	100 ± 2
1 mM PAH	103 ± 4
5 mM Glycine	92.6 ± 4.2
5 mM Proline	90.1 ± 6.2 *
5 mM Leucine	81.4 ± 5.2 **
Control (+20 mM Sodium chloride)	100 ± 2
20 mM Lactate	28.4 ± 0.3 **
20 mM Pyruvate	23.2 ± 1.5 **
20 mM Acetate	29.1 ± 1.1 **
20 mM Propionate	26.7 ± 1.0 **
20 mM Butyrate	25.5 ± 0.5 **
20 mM β-Hydroxybutyrate	22.2 ± 0.9 **
20 mM Nicotinate	27.5 ± 2.8
Control (+0.5% DMSO)	100 ± 2
1 mM Probenecid	45.0 ± 3.3 **

Uptake of HMB (30 μM) in hCMEC/D3 cells was measured in a Na^+^-free buffer, pH 6.0, in the absence (control) or presence of various compounds at indicated concentrations.*, *p* < 0.05 and **, *p* < 0.01 compared to the corresponding control uptake. PAH, *p*-aminohippurate; DMSO, dimethylsulfoxide; HMB, β-hydroxy-β-methylbutyrate.

## Data Availability

All the data relevant to the studies are provided in the manuscript.
